# Altered grey matter structural covariance in chronic moderate–severe traumatic brain injury

**DOI:** 10.1038/s41598-023-50396-7

**Published:** 2024-01-19

**Authors:** Georgia F. Symons, Matthew C. Gregg, Amelia J. Hicks, Christopher C. Rowe, Sandy R. Shultz, Jennie L. Ponsford, Gershon Spitz

**Affiliations:** 1 Department of Neuroscience, Monash University, 6th Floor, The Alfred Centre, 99 Commercial Road, Melbourne, VIC 3004 Australia; 2 Monash-Epworth Rehabilitation Research Centre, Ground Floor, 185-187 Hoddle St, Richmond, 3121 Australia; 3 Department of Molecular Imaging and Therapy, Austin Health, 145 Studley Rd, Heidelberg, VIC 3084 Australia; 4 Health Sciences, Vancouver Island University, 900 Fifth Street, Nanaimo, BC V9R 5S5 Canada

**Keywords:** Trauma, Cognitive neuroscience, Computational neuroscience

## Abstract

Traumatic brain injury (TBI) alters brain network connectivity. Structural covariance networks (SCNs) reflect morphological covariation between brain regions. SCNs may elucidate how altered brain network topology in TBI influences long-term outcomes. Here, we assessed whether SCN organisation is altered in individuals with chronic moderate–severe TBI (≥ 10 years post-injury) and associations with cognitive performance. This case–control study included fifty individuals with chronic moderate–severe TBI compared to 75 healthy controls recruited from an ongoing longitudinal head injury outcome study. SCNs were constructed using grey matter volume measurements from T1-weighted MRI images. Global and regional SCN organisation in relation to group membership and cognitive ability was examined using regression analyses. Globally, TBI participants had reduced small-worldness, longer characteristic path length, higher clustering, and higher modularity globally (*p* < 0.05). Regionally, TBI participants had greater betweenness centrality (*p* < 0.05) in frontal and central areas of the cortex. No significant associations were observed between global network measures and cognitive ability in participants with TBI (*p* > 0.05). Chronic moderate–severe TBI was associated with a shift towards a more segregated global network topology and altered organisation in frontal and central brain regions. There was no evidence that SCNs are associated with cognition.

## Introduction

Moderate to severe traumatic brain injury (TBI) can cause persistent impairments^1^. Cognition is one notable domain, presenting with long-term deficits in attention, processing speed, memory, and executive function^2–7^. These impairments result in sustained disability that limits everyday functioning and reduces quality of life^8^. Developments in neuroimaging have facilitated better characterisation of the underlying mechanisms of persistent deficits in TBI^9^. Aberrant changes in brain network connectivity are now recognised as a critical factor in maintaining persistent deficits, including chronic cognitive impairments^7,10^. The topology of large-scale brain networks may be pathologically altered or reorganised in TBI patients experiencing cognitive impairments^5,11–14^. The extant research is, however, primarily restricted to the first 2 years post-injury, with very few studies assessing brain network disruptions in more chronic periods following TBI.

Structural covariance networks (SCNs) have emerged as a method of investigating the anatomical organisation of large-scale brain networks in neurological disorders such as TBI. This method assesses patterns of covariation in brain morphology between different grey matter regions of interest^15^. Although the exact physiological underpinnings of structural covariance are unknown, SCNs likely reflect a combination of anatomical white matter connections, inter-regional functional connectivity, and synchronised maturational changes between related brain regions^15–17^. Aberrant SCNs are evident in Alzheimer’s disease^18,19^, Parkinson’s disease^20^, obsessive–compulsive disorder^21^, and schizophrenia^22^. More recently, disruptions in structural covariance have been reported in individuals with mild TBI^23^ and paediatric TBI^24,25^. These studies provide preliminary support for the utility of SCNs in TBI research. However, they offer little insight into how brain networks might be disrupted in adults with chronic moderate–severe TBI and were not assessed using a graph theoretical framework.

Graph theory can be used to quantify biologically meaningful topological network properties^26,27^. Networks are characterised by global measures of integration (i.e., path length), segregation (i.e., modularity and clustering) and the balance between these (i.e., small-worldness), as well as regional measures (i.e., betweenness and eigenvector centrality)^28^. Healthy brains generally display small-world network topologies, in which network segregation and integration are balanced through high local clustering and short characteristic path lengths. Small-world networks reflect the optimal organisation for cognitive functioning. They are linked with superior performance on cognitive tests of memory, attention, processing speed, and executive function in healthy participants^29^.

Structural and functional connectivity studies have indicated that small-world network architecture may be disrupted following chronic moderate-to-severe TBI^11–13,30,31^, resulting in organisational differences that alter network integration, segregation, and centrality^32^. Notably, these studies also reported longer path-length^30,31^, reduced centrality^13^ and connectivity^31^ but no change in clustering^12,30,31^. While previous studies have demonstrated altered structural covariance in TBI, these did not use a graph theoretical approach^24,25,33^. The present study, therefore, offers a novel approach to assessing SCNs. Given that SCNs are thought to be related to functional and white-matter connectivity^15–17^ we expect SCNs to resemble network alterations reported in previous chronic moderate-to-severe TBI cohorts^11–13,30,31^.

The primary aim of this study was to use individualised SCNs to examine differences in global and regional brain organisation in individuals at least 10 years after a single moderate–severe TBI relative to demographically similar healthy controls. Secondary to this, we also investigated whether global SCN organisation was associated with cognitive performance. Regional metrics were assessed at 68 cortical grey matter regions based on the Desikan-Killiany parcellation atlas. We hypothesised that individuals with TBI would display significantly longer average path lengths, significantly lower clustering, modularity, and small-worldness in their global SCNs and significantly lower betweenness and eigenvector centrality in their regional SCNs than healthy controls. Furthermore, global network characteristics would be associated with poorer cognition in individuals with TBI.

## Materials and methods

### Standard protocol approvals, registration, and patient consents

Written informed consent was provided by all participants (or their legal guardian) before study procedures. Ethics for this study were approved by the Austin Health Human Research Ethics Committee (HREC/17/Austin/202). All practices were conducted in accordance with the relevant guidelines and regulations approved by the Austin Health Human Research Ethics Committee.

### Participants

This study included 50 participants with chronic-stage TBI (mean time since injury = 22.60 years, *SD* = 5.95 years, *Range* = 10–33 years) aged between 40 and 80 at the time of assessment (68% male; mean age = 56.56 years, SD = 10.65 years). Participants were recruited from an ongoing longitudinal head injury outcome study which included consecutive admissions to a TBI inpatient rehabilitation program^34^. Eligible participants were required to meet the following criteria: (i) sustained a single moderate to severe TBI according to the Mayo classification^35^ at least 10 years prior to study enrolment, (ii) were at least 16 years of age at the time of injury, (iii) were 40 years or older at the time of study enrolment, and (iv) had no visible focal or vascular lesions detected during visual assessment of MR images, that may lead to errors in cortical surface reconstruction^36^.

Seventy-five healthy control participants were recruited from the general community via social media and newspaper advertisements, aged between 40 and 87 (53% male; mean age = 59.31 years, SD = 11.34 years). Control participants were required to satisfy the following criteria: (i) no previous instance of TBI, concussion, or any loss of consciousness due to a head injury, and (ii) aged 40 years or older at the time of study enrolment. One control participant was excluded from cognitive analysis due to incomplete cognitive data.

Participants with a TBI and healthy controls were also required to meet the following criteria: (i) proficiency in English language, cognitive capacity, and overall medical health to complete study measures, (ii) absence of chronic substance abuse or severe psychiatric disturbances, (iii) no presence of other neurological conditions, (iv) no contraindications for MRI.

### Clinical evaluations

Participants completed a research interview to provide demographic information and medical history. Injury-related information (e.g., date of injury, PTA duration) was obtained from medical records—time since injury was defined as the time between the injury date and clinical evaluation date. Participants completed a battery of cognitive tests assessing domains of memory, processing speed, visuospatial ability, and cognitive flexibility. These included Trail Making Test (Part A and Part B), Rey–Osterrieth Complex Figure test, Rey Auditory Verbal Learning Test, Wechsler Memory Scale (Logical Memory I/II subscale), Controlled Oral Word Association Test, Category Verbal Fluency Test, Digit Symbol Coding test, Digit Span (Forwards and Backwards). Pre-morbid intellectual function was measured using the Wechsler Test of Adult Reading (WTAR UK). An overview of neuropsychological assessments and their associated cognitive domain is included in the supplementary information (SI Table 1).

### Image acquisition and pre-processing

T1-weighted structural MRI sequences were acquired for each participant using a Siemens Magnetom Skyra 3-Tesla MRI scanner (Erlangen, Germany), with the following parameters: inversion time (TI) = 900 ms, repetition time (TR) = 1900 ms, echo time (TE) = 2.43 ms, resolution = 256 × 256, flip angle = 9, field of view (FOV) = 256 mm, slice thickness = 1.00 mm (176 slices). The T1-weighted MRI scans were then segmented and normalised to standard spaces using the ‘fMRIprep’ pre-processing pipeline. Cortical surface estimations, comprising the *white matter surface* and the *pial surface*, were generated using FreeSurfer (version 6.0.0; Fischl^37^). To ensure quality control, cortical surface estimations were visually inspected and manually corrected where necessary. The corrected cortical surfaces were inflated and parcellated into 68 (34 left and 34 right) distinct cortical grey matter regions based on the Desikan-Killiany parcellation atlas^38^.

### Structural covariance networks

**Figure 1:**
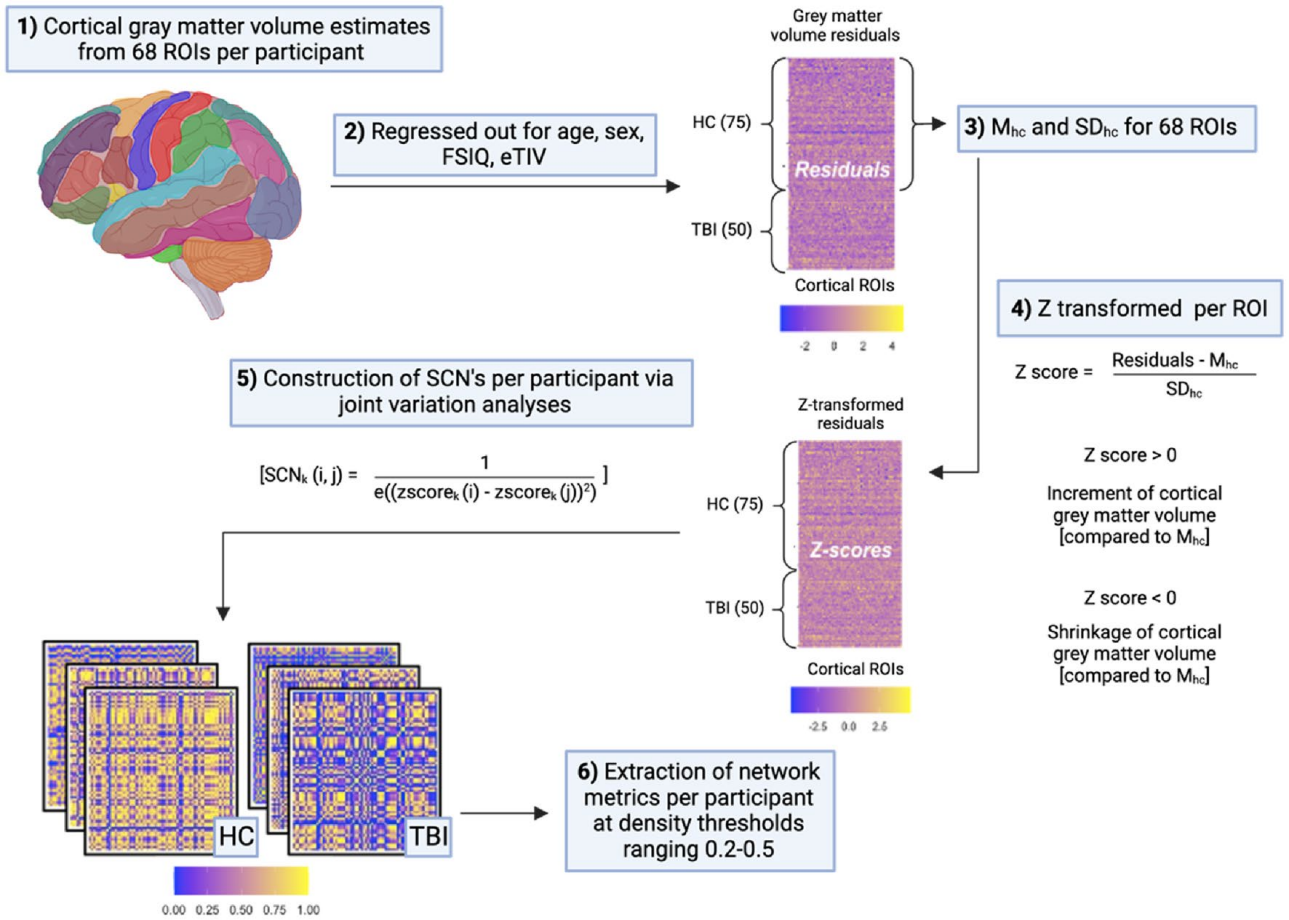
Flowchart depicting the process for constructing participant structural covariance networks. Schematic description of the process for constructing structural covariance networks. *SCN*_*k*_(*i*, *j*) refers to the subject-specific structural covariance (joint variation) between the *i*th (where *i* = 1–68) and *j*th (where *j* = 1–68) ROI for the *k*th (where *k* = 1–125) participant, *zscore*_*k*_(*i*) is the z-transformed value of the *i*th region of grey matter volume for the *k*th participant, and *zscore*_*k*_(*j*) is the z-transformed value of the *j*th region of grey matter volume for the *k*th participant^21^. *ROIs* regions of interest; *FSIQ* full-scale intelligence quotient (WTAR UK), *eTIV* estimated total intracranial volume, *HC* healthy controls, *TBI* traumatic brain injury; *M* mean, *SD* standard deviation. Adapted from Yun et al., (2020).

Intra-individual SCNs were constructed following the methodological pipeline outlined by Yun et al.^21^. This pipeline was implemented using the *brainGraph* package in R (version 3.0.2; Watson^39^). As illustrated in Fig. 1, grey matter volume calculations from 68 cortical regions of interest were corrected for age at assessment, sex, premorbid IQ, and estimated total intracranial volume. The resulting residuals were z-score transformed based on the mean and standard deviation values of each region of interest in the healthy control group. A measure of joint variation between the 68 cortical regions of interest formed the edges of each participant’s individualised SCN. Participant SCNs were thresholded and binarised at network density values ranging from *K* = 0.2–0.5 (with interval 0.1). This process was applied to remove spurious connections from the network by retaining only the strongest edges in the matrix (i.e., a threshold of 0.2 indicates that only the strongest 20% of connections are retained).

#### Global network characteristics

**Figure 2:**
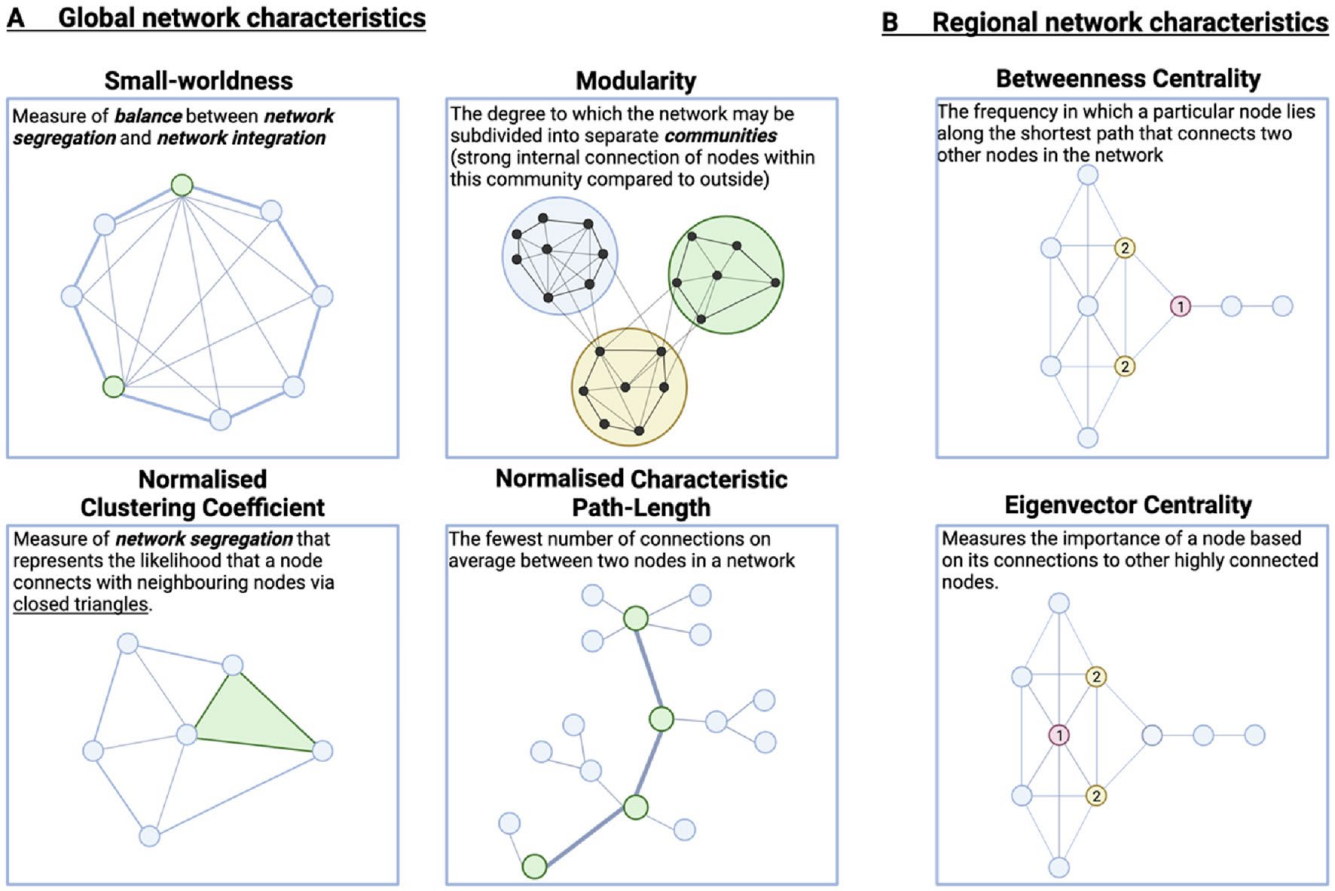
Graphical representation of graph network metrics. Graphical representation of (**A**) global and (**B**) regional graph network metrics assessed in this study. Adapted from Yun et al., (2020).

Four global brain network metrics were extracted from these thresholded and binarised SCNs. These are graphically represented in Fig. 2A and include:

(i) Global modularity; measures the extent to which a network is segregated into separate communities with strong internal connections where nodes are more strongly connected to others within the community than with nodes outside the community.
(ii) Normalised clustering coefficient; is a measure of network segregation representing the likelihood that a node connects with any of its neighbouring nodes to form closed triangles with their edges.
(iii) Normalised characteristic path length; a measure of network integration that denotes the fewest number of connections it takes on average for any two nodes in the network to link.
(iv) Small-worldness; measures the balance between network segregation (i.e. high clustering) and integration (i.e., short characteristic path lengths) of efficient communication within the network.

Our key measure for small-worldness was *ω*^40^. The *ω* index defines small-worldness by comparing each participant’s characteristic path length, *L*, to an equivalent random network, *L*_*rand*_, while comparing their network clustering, *C*, to an equivalent lattice network, *C*_*latt*_, according to the following formula:

1 $$\omega = \frac{{L_{rand} }}{L} - \frac{C}{{C_{latt} }}$$

Refer to Telesford et al.^40^ for full details regarding the calculation and application of the *ω* small-world index.

#### Regional network characteristics

Regional metrics were extracted from participant SCNs to assess morphological network organisation at each of the 68 cortical regions of interest. These regional network metrics are graphically represented in Fig. 2B and include:

(i) Betweenness centrality; the frequency in which a particular node lies along the shortest path that connects two other nodes in the network.
(ii) Eigenvector centrality; a self-referential metric that measures the importance of a node based on its connections to other highly connected nodes. A node’s eigenvector centrality is high if it connects to nodes that are themselves highly connected.

### Statistical analysis

Data analysis was conducted using R (version 4.2.2). All statistical analyses were two-tailed and used a *p* < 0.05 alpha level of significance. Benjamini-Hochberg/false discovery rate corrections were applied to p-values to correct for multiple comparisons of all regression analysis.

A two-sided independent samples t-test was used to determine group-level differences between demographics. Linear regression analyses were conducted to examine the relationship between measures of global SCN organisation (small-worldness, normalised clustering coefficient, normalised characteristic path length, modularity) and group membership (TBI versus control). A series of linear regression analyses were used to explore the association between regional SCN characteristics (betweenness centrality, eigenvector centrality) and group membership at each of the 68 cortical regions defined by the Desikan-Killiany parcellation atlas^38^. Eta squared (η^2^) was used to measure effect size in all analyses that explored group differences in global and regional network characteristics given the categorical nature of the independent variable^41^. Sex, age at assessment, full-scale IQ, and estimated total intracranial volume were controlled for during the creation of participant SCNs and were, therefore not re-used, as covariates in the global and regional regression analyses.

To explore the association between global brain network metrics and cognition, exploratory factor analysis was first conducted to reduce the large battery of cognitive tests into a set of robust factors. Factor analysis was conducted in all participants. An identical factor analysis using only the TBI group that identified the same three factors is included in the Supplementary materials (SI Fig. 1). We used parallel analysis to determine the optimal number of factors to extract and an oblique rotation to allow correlation between factors. Variables with factor loadings of ± 0.32 or above were deemed to have a good factor loading. Bartlett factor scores were derived for each of the extracted factors to assess participants’ individual standings on those factors. In the TBI group alone, each participant's factor scores were modelled as functions of the four global network metrics using linear regression to determine the relationship between cognition and global network organisation. Regression analysis was controlled for sex, age, brain volume and premorbid IQ as these were not previously controlled for with respect to the cognitive measures. Eta squared (η^2^) was used to measure effect size.

All the regression analyses presented in this paper used SCN metrics obtained at a network density threshold of K = 0.2. *K* refers to the network density at which SCNs can be thresholded and binarised and aims to remove spurious network connections while maintaining meaningful connections. The 0.2 threshold value has been used extensively to analyse structural covariance in other neurological disorders^21,42,43^, with research suggesting this threshold lies within the appropriate sparsity range to detect disease-related differences in network connectivity while remaining robust against false positive and false negative connections^44–46^.

Identical experimental thresholding was conducted at K = 0.3–0.5 (with interval of 0.1). A graphical representation of global network metrics at different thresholds can be found in supplementary materials (SI Fig. 2). Additional regression analysis at a global network level was conducted at K = 0.2–0.5 to confirm there was no effect of thresholding on the relationship between network connectivity and our variables of interest (SI Table 2).

## Results

### Demographic information

TBI participants (68% male; mean age = 56.56 years, SD = 10.65 years) did not significantly differ from control participants (53% male; mean age = 59.31 years, SD = 11.34 years) with respect to age and sex (*p* = 0.177 & *p* = 0.1000 respectively). Control participants (Mean = 105.61, SD [Range] = 7.57 [84–117]) did, however, have significantly greater scores on the Wechsler Test of Adult Reading a measure of pre-morbid IQ compared to TBI participants (Mean = 101.42, SD [Range] = 8.06 [87–116]) (*p *= 0.004).

### Global network organisation following TBI

**Figure 3:**
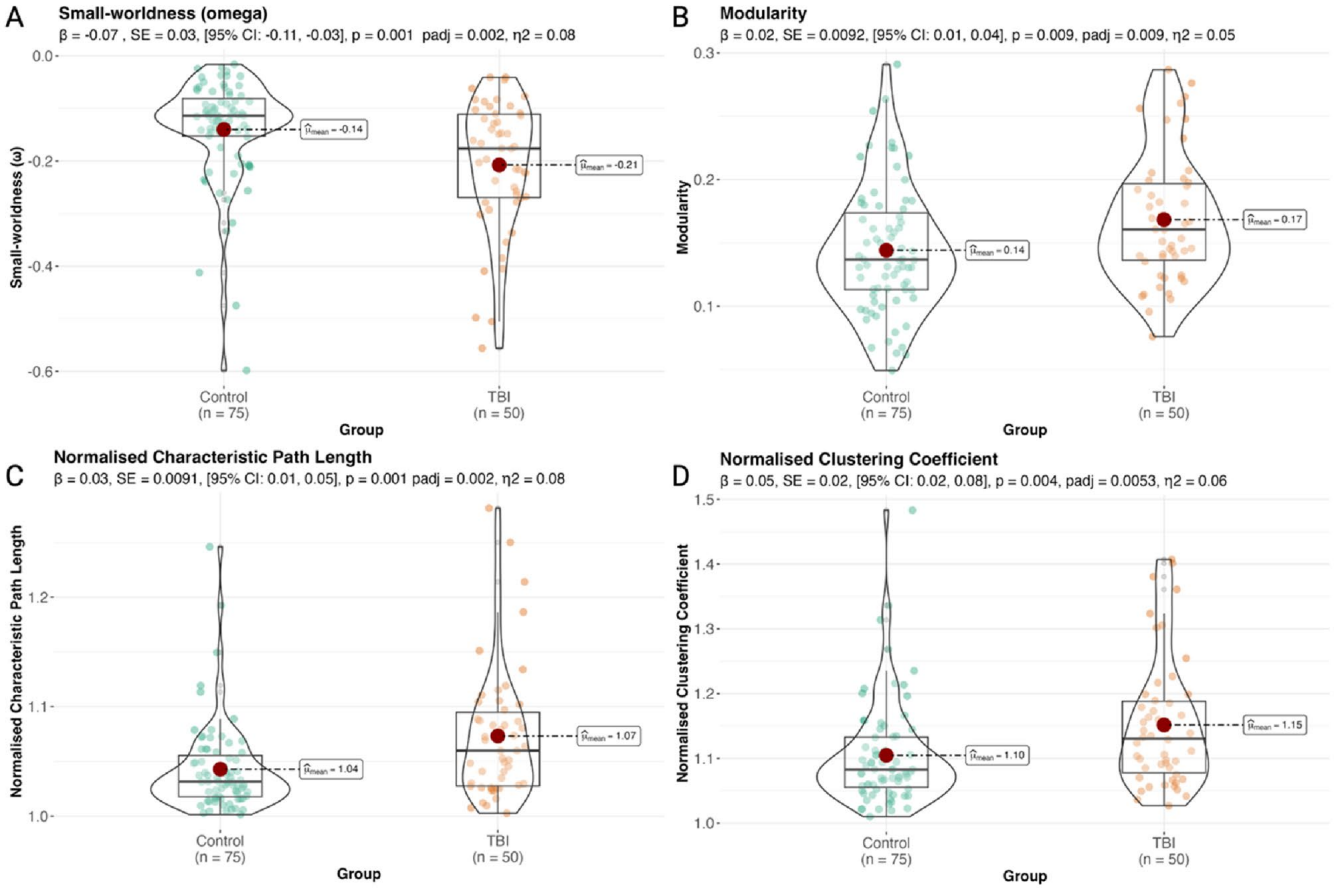
TBI participants displayed impaired global network characteristics compared to controls. Figure depicts boxviolin plots to compare group differences in four key global network characteristics. Significant associations were found between TBI group membership and (**A**) lower small-worldness (values closer to zero are indicative of greater small-worldness) (**B**) longer normalised characteristic path length, (**C**) higher normalised clustering, and (**D**) higher modularity following linear regression analysis indicative of a more segregated global network topology compared to controls. β = Beta coefficients, *p* = *p*-values, padj = *p*-values adjusted using the Benjamini & Hochberg method, 95% *CI* confidence intervals; *l* left; *r* right; *SE* standard error, n^2^ = eta squared.

As hypothesised, small-worldness (*ω*) was significantly lower in TBI participants compared to healthy controls (*β* = − 0.07, *SE* = 0.02, [95% CI − 0.11, − 0.03], *p*_adj_ = 0.002, *η*^2^ = 0.08; Fig. 3A). TBI group membership was also significantly associated with higher modularity (*β* = 0.02, *SE* = 0.0092, [95% CI 0.01, 0.04], *p*_adj_ = 0.009, *η*^2^ = 0.05; Fig. 3B), longer normalised characteristic path lengths (*β* = 0.03, *SE* = 0.00904, [95% CI 0.01, 0.05], *p*_adj_ = 0.002, *η*^2^ = 0.08; Fig. 3C) and higher normalised clustering (*β* = 0.05, *SE* = 0.02, [95% CI 0.02, 0.08], *p*_adj_ = 0.0053, *η*^2^ = 0.06; Fig. 3D). These findings remained consistent when regression analyses were run at each network density threshold *K* = 0.2–0.5 (with increment 0.1; SI Table 2).

### Regional network organisation following TBI

**Figure 4:**
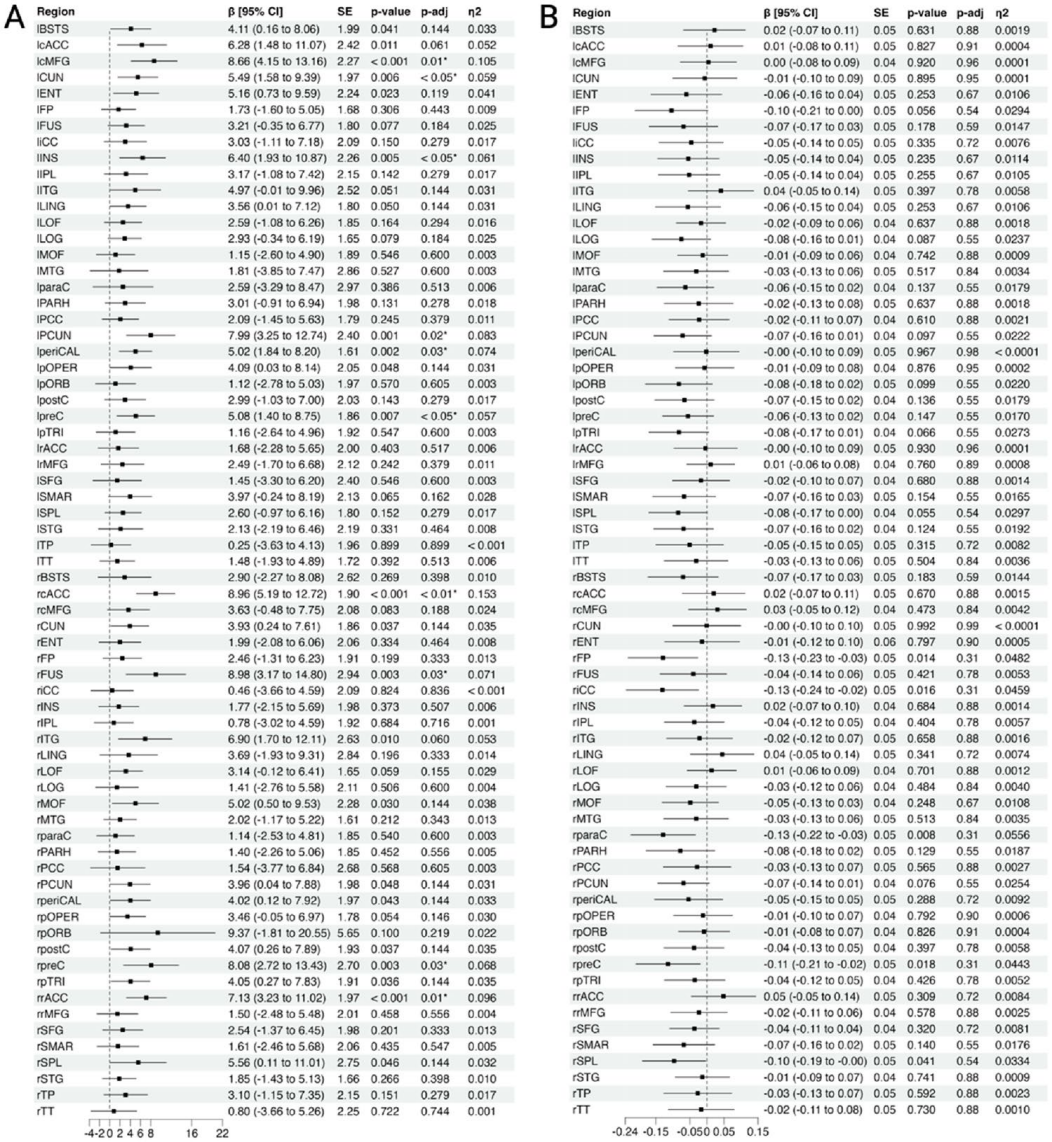
Group differences in betweenness centrality and eigenvector centrality at each cortical region. (**A**) Participants with TBI had significantly greater betweenness centrality in 10 cortical regions after adjusting for false discovery rate (indicated by *). These regions are presented graphically in Fig. 5. (**B**) No significant differences were found in eigenvector centrality between individuals with TBI and healthy controls at any cortical region. This figure plots the regression coefficients with 95% confidence intervals, where a positive β coefficient indicates higher betweenness or eigenvector centrality in the TBI group compared to healthy controls. *p*-value = unadjusted pvalues; padj = *p*-values adjusted using the Benjamini–Hochberg method, *CI* confidence intervals; *l* left; *r* right; *SE* standard error, n^2^ = eta squared.

**Figure 5:**
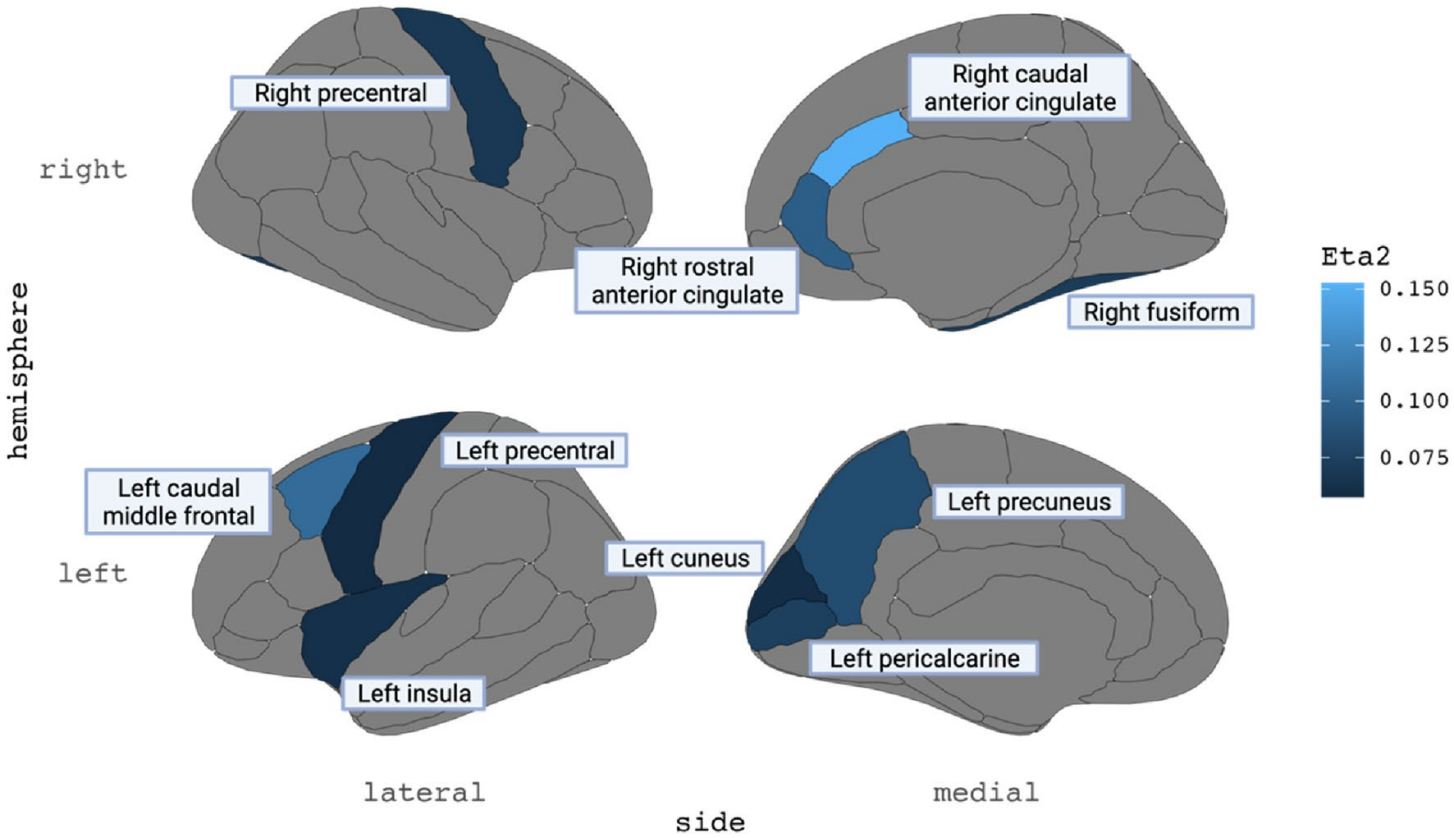
Greater betweenness centrality is associated with TBI group membership in selected cortical regions. Graphical representation of cortical regions where betweenness centrality was significantly higher in TBI participants compared to healthy controls following false discovery rate corrections (Fig. 4A). Regions are highlighted based on their effect sizes (Eta2 = η^2^). Lighter colours reflect a larger effect size.

The relationship between group membership regional network measures (betweenness centrality and eigenvector centrality) was assessed at 68 distinct cortical regions using a series of linear regression analyses (Fig. 4). Compared to healthy controls, TBI participants were found to have significantly higher betweenness centrality in 10 of the 68 cortical regions investigated after correcting for multiple comparisons (Fig. 4A). There was a significant association between group membership and eigenvector centrality in five regions; however, this did not hold following adjustment for multiple comparisons (Fig. 4B). The regions with significantly greater betweenness centrality are depicted graphically in Fig. 5.

### Global brain network organisation and cognitive outcomes

**Figure 6:**
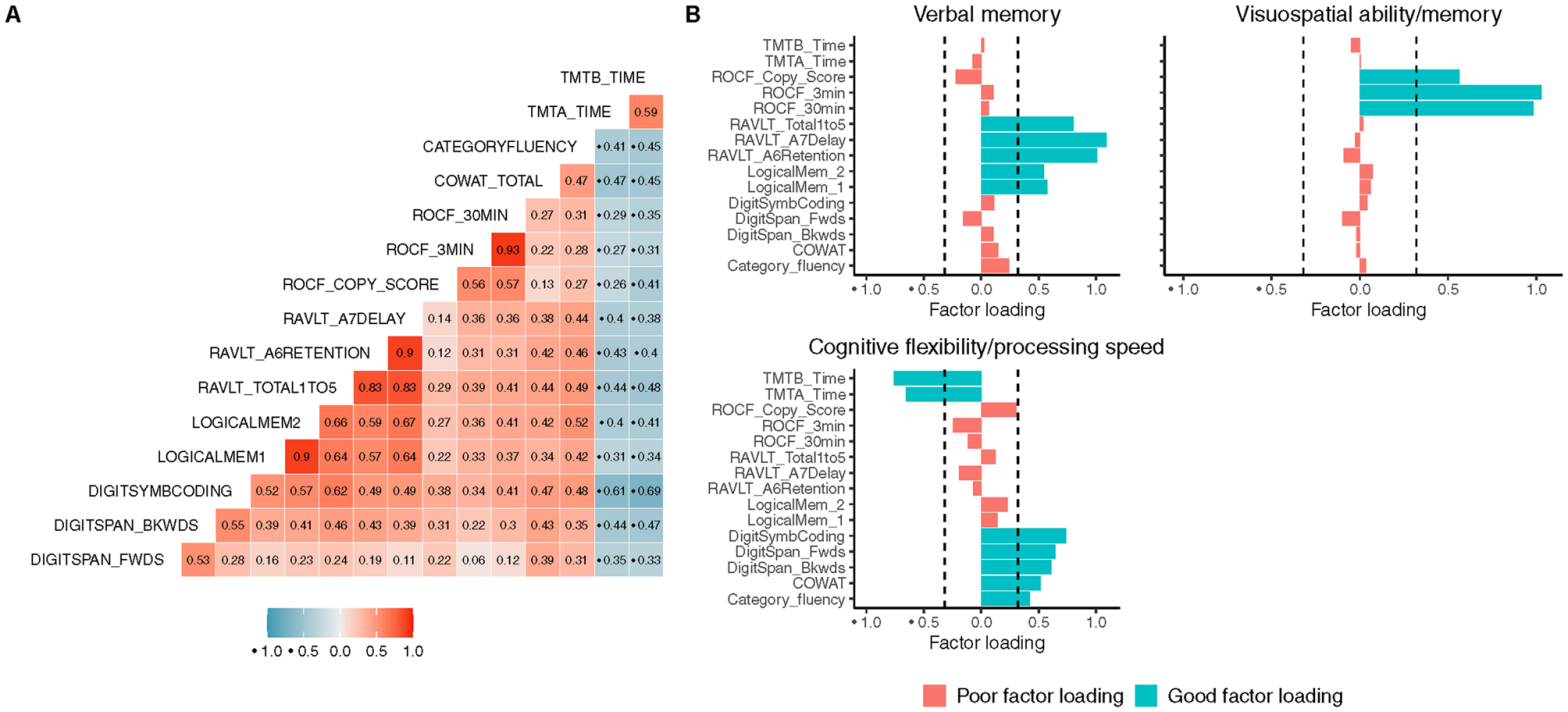
Factor analysis for neuropsychological test domain reduction. Results of exploratory factor analysis on neuropsychological tests for all participants. (**A**) Correlation matrix between each neuropsychological measure. Colour bar represents the strength of correlation (**B**) The three factors extracted from exploratory factor analysis. Bar plots depict the loading of each variable on the factors. Bars are colour-coded based on whether they display a factor loading of ± 0.32 or above, where variables that load most strongly on a factor are coloured blue. Abbreviations: TMTB_Time = Trail Making Test-Part B; TMTA_Time = Trail Making Test-Part A; ROCF_Copy_Score = Rey–Osterrieth Complex Figure test, copy score; ROCF_3min = Rey–Osterrieth Complex Figure test, 3 min delay; ROCF_30min = Rey–Osterrieth Complex Figure test, 30 min delay; RAVLT_Total1to5 = Rey Auditory Verbal Learning test, list 1–5 learning; RAVLT_A6Retention = Rey Auditory Verbal Learning test RAVLT_A7Delay = Rey Auditory Verbal Learning test, delay; retention; LogicalMem_1 = Wechsler Memory Scale Logical Memory I; LogicalMem_2 = Wechsler Memory Scale Logical Memory II Delay condition;; DigitSymbCoding = Digit Symbol Coding test; DigitSpan_FWDS = Digit Span, Forwards; DigitSpan_BKWDS = Digit Span, Backwards; COWAT_TOTAL = Controlled Oral Word Association Test; Category_fluency = Category Verbal Fluency Test.

We aimed to examine the relationship between global network characteristics and cognition within the TBI group. Whilst not the main aim of the present study, at a group level, cognitive performance differed on several neuropsychological tests (SI Table 3). Exploratory factor analysis using all participants was conducted. A parallel analysis using all participants identified three factors (SI Fig. 3) *verbal memory*, *visuospatial ability and memory*, and *cognitive flexibility/processing speed* (Fig. 6). Factor scores were extracted for each participant to determine their individual standing on the three factors. Factor scores were also assessed at a group level (SI Table 3).

**Figure 7:**
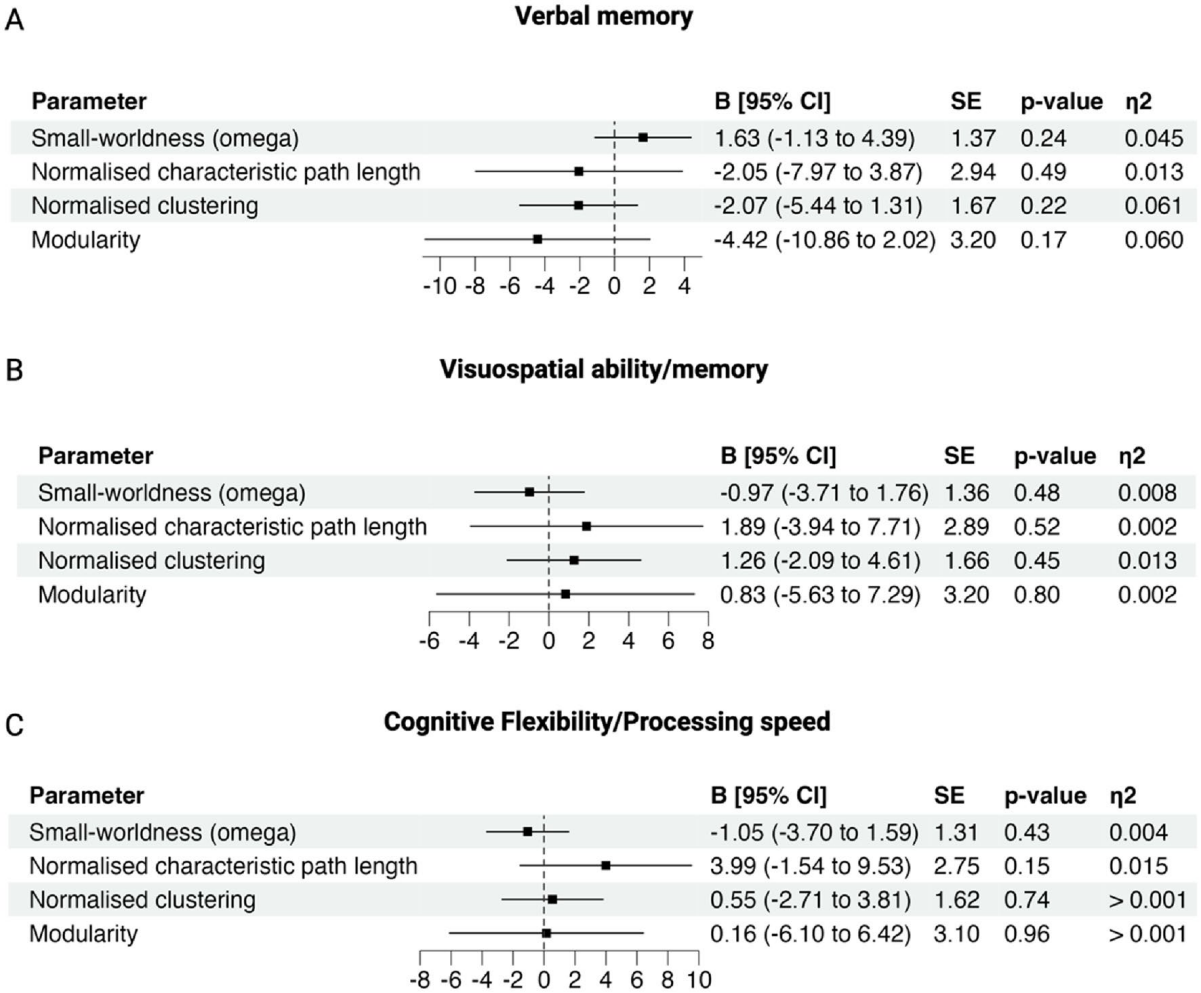
Association between global brain characteristics and cognitive performance in TBI participants. Forrest plot of the regression coefficients with 95% confidence intervals. Global brain network characteristics were not associated with (**A**) verbal memory (**B**) visuospatial ability/memory or (**C**) cognitive flexibility/processing speed in the TBI group. *CI* confidence intervals, *SE* standard error, n^2^ = eta squared.

In the TBI group alone, we regressed these factor scores against each participant’s global network characteristics to determine whether global brain network organisation was associated with specific cognitive domains. Regressions were controlled for sex, age at assessment, and premorbid IQ. There was no significant association between global network characteristics and visuospatial ability/memory, verbal memory, or cognitive flexibility/processing speed (all regressions *p* > 0.05; Fig. 7). These analyses were replicated in control participants and depicted in the supplementary information (SI Fig. 4).

## Discussion

Moderate to severe TBI causes significant long-term morbidity contributing to poor quality of life. Brain network reorganisation may contribute to impairments; however, little is known, particularly in the chronic period following TBI. The current study investigated SCNs that reflect morphological covariation between brain regions. The primary aim of this study was to characterise the global (small-worldness, characteristic path length, clustering, and modularity) and regional (betweenness centrality and eigenvector centrality) brain network characteristics in SCNs in the chronic period (≥ 10 years post-injury) following a single moderate to severe TBI. The secondary aim was to determine whether global network organisation was associated with cognitive performance. Compared to healthy controls, TBI participants had a global reduction in small-worldness and increased normalised clustering, modularity, and normalised characteristic path length. Regionally, TBI group membership was associated with reduced betweenness centrality in ten frontal and central cortical regions. No significant differences were identified in eigenvector centrality. There was no association between global network measures and cognitive performance in the TBI group.

### Global network

At the global network level, we observed reduced small-worldness and increased normalised clustering, modularity, and normalised characteristic path lengths in the TBI SCNs compared with healthy control networks. Notably, the directionality of change for small-worldness and path length, but not modularity and clustering, was consistent with our hypothesis. These alterations are indicative of a shift towards a segregated lattice global SCN organisation in individuals with TBI. Small-worldness reflects an optimal balance between regional segregation and global integration. Comparatively, the lattice network structure relies more heavily on local specialisation, thus reducing global efficiency and network integration across the brain^47,48^. Our results suggest that the intricate network architecture of the brain is disrupted following a single moderate to severe TBI, and such alterations persist in the chronic post-injury period. Consistent with our findings, several studies have reported similar lattice network properties in moderate to severe TBI samples derived from structural and functional brain network analyses^12,49^. We expand on this past research by mapping brain network connectivity using grey matter structural covariance for the first time in a chronic (≥ 10 years post-injury) moderate to severe TBI sample. Thus, the present study provides evidence of global network segregation in the years following a single moderate to severe TBI.

We did not observe a global decrease in modularity and clustering coefficient as hypothesised. Yuan et al.^51^ suggested that global brain networks may become more segregated in children with mild TBI to compensate for damaged long-range connections throughout the network. Here, we propose that this compensatory increase in global clustering and modularity may extend to adults in the chronic period following a single moderate to severe TBI as found here. Another explanation is the possibility that greater network segregation is more costly than compensatory^32^, instead reflecting the vulnerability of long-range connections to trauma-related axonal damage and neurodegeneration following TBI. Longitudinal data are required to test these hypotheses and determine the functional implications of a lattice network shift over time.

### Regional network

At the regional network level, we found greater betweenness centrality in individuals with chronic TBI compared with healthy controls in ten cortical regions. These regions were primarily localised to frontal and central brain regions, including the cingulate cortex, precentral gyri, and the middle frontal gyrus. Interestingly, this did not align with our hypothesis. Furthermore, we did not detect significant group differences in eigenvector centrality following false discovery rate corrections. Although little is known regarding the precise effects of TBI on SCNs, our results may reflect disruptions to peripheral connections in these key hub regions of the brain networks. When peripheral connections are disturbed, a greater number of shortest paths must pass through highly connected hub nodes, thereby increasing betweenness centrality at the region. This hypothesis would further explain why no group differences were observed in eigenvector centrality between TBI patients and healthy controls, as eigenvector centrality remains unaffected when neighbouring hubs suffer only peripheral disconnections. Alternatively, it could be that betweenness centrality was increased in TBI patients due to the formation of new, perhaps compensatory, connections that pass through the network’s central hub nodes. If true, adding these hub connections should theoretically increase eigenvector centrality at neighbouring nodes. However, no such effect was present in our TBI sample.

These centrality findings contrast with previous structural connectivity studies that reported reductions in both betweenness centrality and eigenvector centrality in similar frontal and central brain regions of mild and moderate–severe TBI patients with diffuse axonal injury at time points between 4 days and 3 years post-injury^13,50,51^. The reasons for these discrepancies are not immediately evident. One possible explanation is that hub node connections may have a greater capacity for recovery following TBI than peripheral node connections. Unlike previous work, the TBI sample used in the present study comprised of only individuals in the chronic post-injury period. This allows our study to better detect new network connections that might have formed in the years following moderate to severe TBI. Therefore, while TBI may initially reduce network centrality by damaging connections to popular hub nodes, these connections may partially recover and increase network centrality over time.

### Association of network characteristics with cognitive outcomes

Our secondary aim was to investigate the association between global network characteristics and cognitive outcomes in the TBI group. In discordance with our hypothesis, we found no significant associations between the extent of global network structure differences and cognitive functioning in individuals with chronic moderate to severe TBI. Cognitive deficits in domains such as verbal memory and cognitive flexibility have consistently been reported in chronic and moderate to severe samples^2,5,52^ with some studies suggesting that reduced global network efficiency may be implicated^11,13,53^. While we similarly identified significantly worse cognitive performance at a group level in TBI participants compared to healthy controls (SI Table 3), no significant association exists between global structural covariance metrics and cognitive performance. Notably, previous associations between cognitive performance and network metrics were identified in structural connectivity networks. The relative infancy of global structural covariance research in moderate to severe TBI samples, particularly those in the chronic post-injury period, makes it difficult to compare these findings with previous research.

We did not find a relationship between brain network topology and cognition in the present study for several possible reasons. First, genetic or environmental factors (i.e., such as cognitive reserve) may offer some individuals a degree of cognitive resilience to differences in global brain network organisation following TBI. Examining the impact of genetic and environmental factors on the association between SCNs and cognitive function presents a novel avenue for future research, as they may better predict cognitive functioning in the chronic injury period than network organisational differences alone. Second, our chronic TBI sample may have developed personalised strategies in the years following their injuries to help mitigate the influence of brain network alterations on their cognitive functioning as a result of exposure to extensive inpatient and outpatient cognitive rehabilitation, as previously described^34^. Third, the global network metrics used in our study may have been too general to predict performance in specific cognitive domains. Instead, cognitive functioning in the chronic phase following moderate to severe TBI may be better investigated at a regional network level. Furthermore, previous studies that have identified a relationship between network metrics and cognitive performance were conducted in children^54^, sub-acute time points^55,56^ or in the context of white matter structural connectivity^11,13,53,57^; there remains a paucity of this research in SCNs.

### Limitations and future directions

Interestingly, the directionality of differences in global and regional morphological networks in patients in the chronic period following TBI, differed from those hypothesised. Notably, the discrepancy between our findings and previous research could also reflect an important limitation of SCNs. As we do not yet understand the physiological mechanisms that drive differences in structural covariance, SCNs may be affected differently by chronic TBI than white matter networks, resulting in contrasting centrality patterns. Furthermore, the pathological underpinnings of SCN and cognitive changes may differ, thus explaining this lack of association. Future research should focus on a better understanding of the biological basis of structural covariance. Furthermore, the exclusion of TBI patients with large focal brain lesions contributed to selection bias in our sample. While these patients may have demonstrated more extensive brain network differences, the present study notably establishes evidence of structural covariance differences irrespective of large lesions.

## Conclusion

In conclusion, we found that SCNs are reorganised in the chronic period (≥ 10 years post-injury) following a single moderate to severe TBI. Our results suggest that brain network organisation becomes more segregated in the years following injury, with more connections passing through frontal and central hub regions. However, we found no evidence that increased global network segregation was associated with poorer cognitive functioning in chronic TBI. These findings offer insight into how moderate to severe TBI alters brain network connectivity over many years, providing a foundation for future research to explore the physiological basis and functional implications of structural covariance disruption following TBI.

### Supplementary Information

Supplementary Information.

## Data Availability

The data and scans from this study will be made available in a de-identified format to researchers via the Federal Interagency Traumatic Brain Injury Research (FITBIR) database (https://fitbir.nih.gov/).
