# Supplementary material for: Altered grey matter structural covariance in chronic moderate–severe traumatic brain injury
Source: Sci Rep. 2024 Jan 19;14:1728. doi: 10.1038/s41598-023-50396-7 (PMC10799053; doi:10.1038/s41598-023-50396-7)
Supplement: Supplementary file 1 — Supplementary Information. [file 41598_2023_50396_MOESM1_ESM.docx]

**Supplementary Information**

**Title: Altered grey matter structural covariance in chronic moderate-severe traumatic brain injury**

Georgia F. Symons^1#*^, Matthew C. Gregg^2#^, Amelia J. Hicks^2^, Christopher C. Rowe^3^, Sandy R. Shultz^1,4^, Jennie L. Ponsford^2^, Gershon Spitz^1,2^

Author affiliations:

^1^Department of Neuroscience, Monash University, 6th Floor, The Alfred Centre, 99 Commercial Road, Melbourne, VIC 3004, Australia.

^2^Monash-Epworth Rehabilitation Research Centre, Ground Floor, 185-187 Hoddle St, Richmond, 3121, Australia.

^3^Department of Molecular Imaging & Therapy, Austin Health, 145 Studley Rd, Heidelberg VIC 3084, Australia.

^4^Health Sciences, Vancouver Island University, 900 Fifth Street Nanaimo, BC, V9R 5S5, Canada

#Authors contributed equally

**Supplementary Table 1: List of neuropsychological measures**

| Neuropsychological measure | Cognitive domain assessed |
| --- | --- |
| Digit span, Forward | Attention |
| Digit Span, Backwards | Working memory |
| Digit symbol coding | Processing speed/cognitive flexibility |
| Logical Memory 1 | Episodic memory |
| Logical Memory 2 | Episodic memory |
| RAVLT 1 to 5 total | Verbal memory |
| RAVLT A6 Retention | Verbal memory |
| RAVLY A7 Delay | Verbal memory |
| ROCF Copy | Visual memory |
| ROCF 3min | Visual memory |
| ROCF 30min | Visual memory |
| Controlled oral word association test | Cognitive control/executive function |
| Category Verbal Fluency Test | Language |
| Trail making test, Part A | Processing speed |
| Trail making test, Part B | Executive function/set shifting |

**Supplementary Figure 1.** **Exploratory factor analysis on neuropsychological tests for participants with TBI only.**

**(A)** Correlation matrix between each neuropsychological measure. Colour bar represents the strength of correlation **(B)** The three factors extracted from exploratory factor analysis. Bar plots depict the loading of each variable on the factors. Bars are colour-coded based on whether they display a factor loading of $\pm0.32$ or above, where variables that load most strongly on a factor are coloured blue. Abbreviations: TMTB_Time = Trail Making Test–Part B; TMTA_Time = Trail Making Test–Part A; ROCF_Copy_Score = Rey-Osterrieth Complex Figure test, copy score; ROCF_3min = Rey-Osterrieth Complex Figure test, 3min delay; ROCF_30min = Rey-Osterrieth Complex Figure test, 30min delay; RAVLT_Total1to5 = Rey Auditory Verbal Learning test, list 1-5 learning; RAVLT_A6Retention = Rey Auditory Verbal Learning test RAVLT_A7Delay = Rey Auditory Verbal Learning test, delay; retention; LogicalMem_1 = Wechsler Memory Scale Logical Memory I; LogicalMem_2 = Wechsler Memory Scale Logical Memory II Delay condition; DigitSymbCoding = Digit Symbol Coding test; DigitSpan_FWDS = Digit Span, Forwards; DigitSpan_BKWDS = Digit Span, Backwards; COWAT_TOTAL = Controlled Oral Word Association Test; Category_fluency = Category Verbal Fluency Test.

**
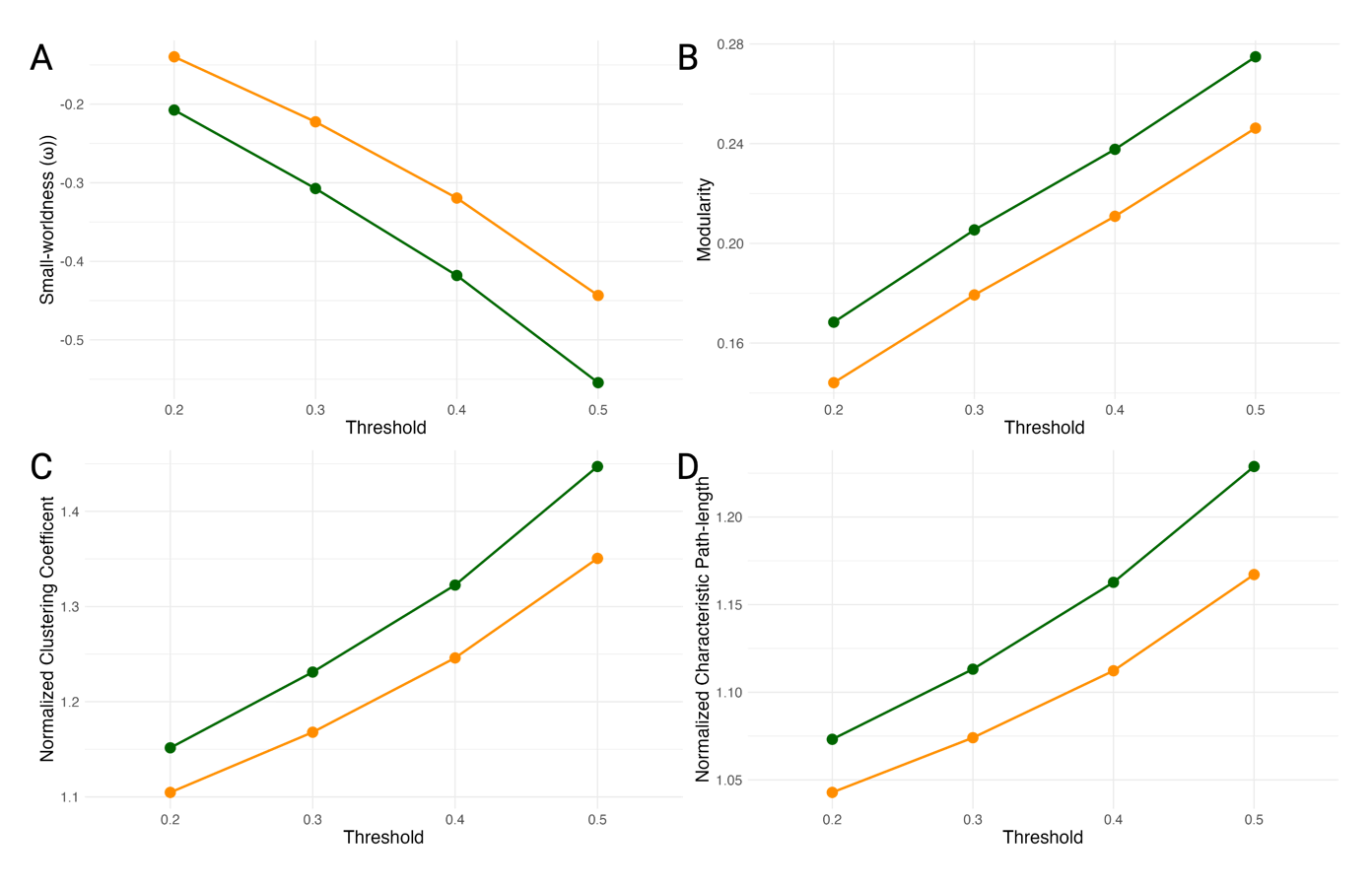
**

**Supplementary Figure 2.** Mean value for **(A)** Small-worldness, **(B)** Modularity, **(C)** Normalized clustering coefficient at thresholds **(D)** Normalized characteristic path length, K = 0.2 – 0.5 (0.1 increments)**.** Green depicts TBI and orange depicts control group membership.

**Supplementary Table 2: Linear regression analyses at different thresholds.**

| Threshold (*K*) | Regression analysis output | | | | |
| --- | --- | --- | --- | --- | --- |
|  | *β* | *SE* | *95% CI* | *p* | *η^2^* |
|  | **Modularity** | | | | |
| 0.2 | 0.02 | 0.0092 | [0.01, 0.04] | 0.009 | 0.05 |
| 0.3 | 0.03 | 0.01 | [0.01, 0.05] | 0.010 | 0.05 |
| 0.4 | 0.03 | 0.01 | [0.01, 0.05] | 0.010 | 0.05 |
| 0.5 | 0.03 | 0.01 | [0.01, 0.05] | 0.009 | 0.05 |
|  | **Normalized Clustering Coefficient** | | | | |
| 0.2 | 0.05 | 0.02 | [0.01, 0.08] | 0.004 | 0.06 |
| 0.3 | 0.06 | 0.02 | [0.02, 0.11] | 0.005 | 0.06 |
| 0.4 | 0.08 | 0.03 | [0.02, 0.13] | 0.007 | 0.06 |
| 0.5 | 0.10 | 0.04 | [0.03, 0.17] | 0.008 | 0.06 |
|  | **Normalized Characteristic Path-length** | | | | |
| 0.2 | 0.03 | 0.00904 | [0.01, 0.05] | 0.001 | 0.08 |
| 0.3 | 0.04 | 0.01 | [0.02, 0.06] | 0.001 | 0.08 |
| 0.4 | 0.05 | 0.01 | [0.02, 0.08] | < .001 | 0.09 |
| 0.5 | 0.06 | 0.02 | [0.03, 0.10] | < .001 | 0.10 |
|  | **Small-worldness (*ω*)** | | | | |
| 0.2 | -0.07 | 0.02 | [-0.11, -0.03] | 0.001 | 0.08 |
| 0.3 | -0.08 | 0.03 | [-0.14, -0.03] | 0.002 | 0.08 |
| 0.4 | -0.10 | 0.03 | [-0.16, -0.04] | 0.002 | 0.08 |
| 0.5 | -0.11 | 0.04 | [-0.18, -0.04] | 0.002 | 0.07 |

Table represents linear regression analysis to examine the association between global network metrics and group membership (TBI vrs controls). At all thresholds, TBI group membership was significantly associated with global network metrics. *p* = unadjusted *p*-values.

**Supplementary Table 3: Cognitive data between TBI and control participants**

|  | TBI | | Controls | | P-value |
| --- | --- | --- | --- | --- | --- |
|  | **Mean** | **SD [range]** | **Mean** | **SD [range]** |  |
| N | 50 |  | 74 |  |  |
| Digit span, Forward | 10.46 | 2.38 [4 – 15] | 10.92 | 2.50 [6 – 16] | 0.309 |
| Digit Span, Backwards | 6.62 | 2.47 [3 – 13] | 7.45 | 2.42 [2 – 13] | 0.067 |
| Digit symbol coding | 61.04 | 20.83 [25 – 111] | 68.12 | 17.01 [17 – 98] | 0.019* |
| Logical Memory 1 | 12.00 | 4.45 [0 – 20] | 13.76 | 3.97 [2 – 22] | 0.023* |
| Logical Memory 2 | 10.20 | 4.70 [0 – 19] | 12.54 | 4.19 [1 – 21] | 0.004* |
| RAVLT 1 to 5 total | 45.94 | 11.67 [19 – 69] | 52.19 | 9.81 [26 – 71] | 0.002* |
| RAVLT A6 Retention | 8.36 | 3.58 [0 – 15] | 10.91 | 2.93 [3 – 15] | < 0.001* |
| RAVLY A7 Delay | 8.16 | 3.96 [0 – 15] | 10.80 | 2.87 [3 – 15] | < 0.001* |
| ROCF Copy | 32.01 | 4.41 [15.5 – 36.0] | 32.51 | 4.10 [18.0 -36.0] | 0.522 |
| ROCF 3min | 16.91 | 6.65 [4.5 – 28.5] | 17.26 | 6.72 [4.0 – 31.5] | 0.773 |
| ROCF 30min | 16.19 | 6.93 [0.00 – 35.00] | 17.24 | 6.58 [3.0 – 34.0] | 0.397 |
| Controlled oral word association test | 38.64 | 10.87 [19 – 60] | 41.80 | 10.91 [20 – 64] | 0.116 |
| Category Verbal Fluency Test | 20.70 | 5.51 [5 – 30] | 23.55 | 5.81 [10 – 44} | 0.007* |
| Trail making test, Part A | 35.46 | 21.74 [12 – 116] | 25.66 | 10.56 [6 – 72] | 0.004* |
| Trail making test, Part B | 87.68 | 58.77 [23 – 314] | 67.77 | 48.22 [28 – 400] | 0.050 |
| Factor 1: Verbal memory | -0.44 | 1.11 [-2.99 – 1.48] | 0.30 | 0.83 [-2.01 – 1.66] | < 0.001* |
| Factor 2: Visuospatial ability/memory | -0.722 | 1.04 [-2.12 – 2.33] | 0.0488 | 1.00 [-2.23 – 2.33] | 0.518 |
| Factor 3: cognitive flexibility/processing speed | -0.30 | 1.24 [-3.95 – 2.18] | 0.21 | 0.88 [-2.60 – 1.78] | 0.014* |

Table depicts the means and standard deviation. A two-sided independent samples t-test was used to determine group-level differences in neuropsychological assessments and factor analysis scores. **Abbreviations:** RAVLT = Rey Auditory Verbal Learning test**,** ROCF = Rey-Osterrieth Complex Figure test.


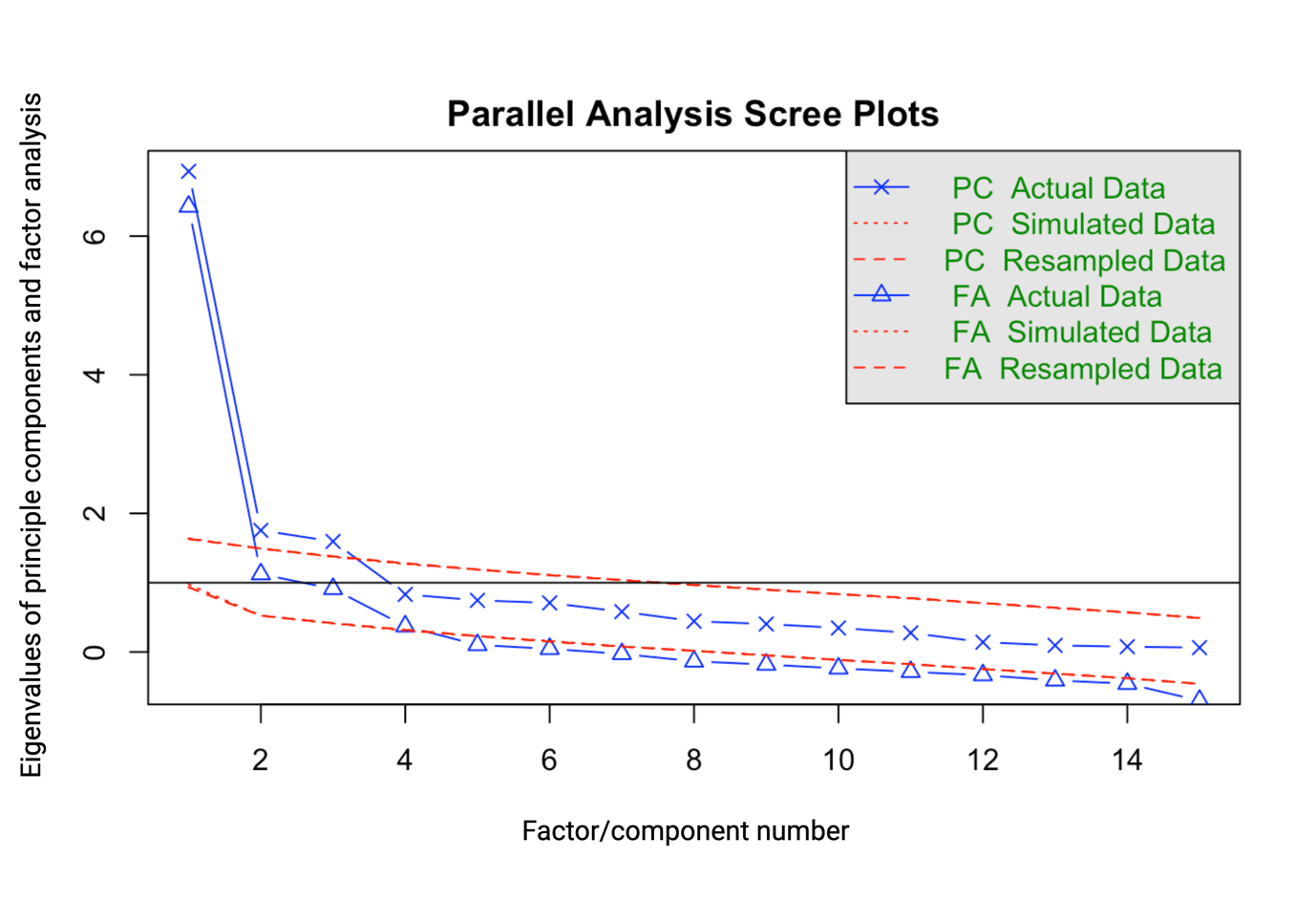
**Supplementary Figure 3.** Parallel Analysis Scree plot for factors analysis depicted in Figure 6.

**
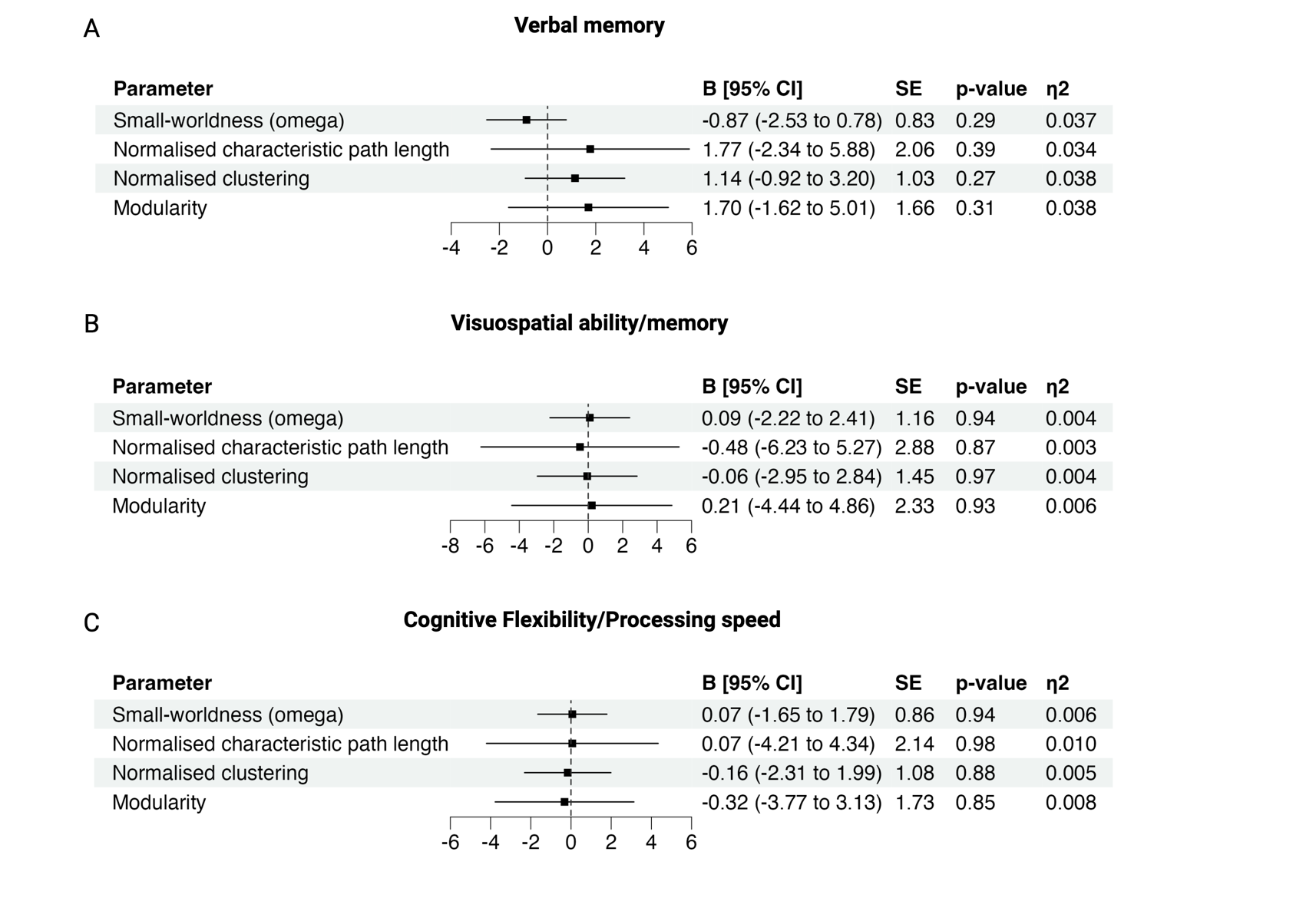
**

**Supplementary Figure 4. Association between global brain characteristics and cognitive performance in control participants.**

Global brain network characteristics were not associated with visuospatial ability/memory **(A)** verbal memory **(B)** visuospatial ability/memory or **(C)** cognitive flexibility/processing speed in the control group.
